# Gastroprotective effect of *Benincasa hispida* fruit extract

**DOI:** 10.4103/0253-7613.45154

**Published:** 2008

**Authors:** Manish A. Rachchh, Sunita M. Jain

**Affiliations:** Department of pharmacology, S. J. Thakkar Pharmacy College, Rajkot, Gujarat, India; 1 Department of pharmacology, L. M. College of Pharmacy, Ahmedabad, Gujarat, India

**Keywords:** Antiulcer, antioxidant, ulcer index, vascular permeability

## Abstract

**Objectives:**

The antiulcer activity of *Benincasa hispida* (Thunb.) Cogn. fruit was evaluated in rats against ethanol-induced gastric mucosal damage, pylorus ligated (PL) gastric ulcers, and cold restraint-stress (CRS)-induced gastric ulcer models.

**Methods:**

Petroleum ether and methanol extracts were administrated orally at the dose of 300 mg/kg, and omeprazole (reference standard) at the dose of 20 mg/kg. Ulcer index was common parameter studied in all the models. Further, vascular permeability was evaluated in ethanol model, and effect on lipid peroxidation, viz. melondialdehyde (MDA) content, superoxide dismutase (SOD), and catalase (CAT) levels were studied in CRS model.

**Results:**

Both the extracts produced significant reduction in ulcer index (*P* < 0.05) in all the models and the results were comparable with that of omeprazole-treated group. Further, significant reduction in vascular permeability (*P* < 0.05) was observed. In CRS model, MDA content was significantly reduced along with increase in CAT levels as compared to control group.

**Conclusions:**

Petroleum ether and methanol extracts of *B. hispida* possess significant antiulcer as well as antioxidant property.

## Introduction

*Benincasa hispida* (Thunb) Cogn. (Family: Cucurbitaceae) is commonly known as *Bhuru Kolu* or *Safed Kolu* (Gujarati), *Petha* (Hindi), white pumpkin or wax gourd or ash gourd (English), and *Kushmanda* (Sanskrit). Fruits of this plant are traditionally used as a laxative, diuretic, tonic, aphrodisiac, cardiotonic, urinary calculi, blood disease, insanity, epilepsy, and also in cases of jaundice, dyspepsia, fever, and menstrual disorders.[1]

The methanolic extract of the fruit is reported to possess antiulcer,[2] anti-inflammatory,[3] antihistaminic, and antidepressant activities.[4] Phytochemical review indicates the presence of triterpenes: alnusenol, multiflorenol, iso-multiflorenol; flavone: iso-vitexin; and sterols: lupeol, lupeol acetate, and beta-sitosterol.[5]

A number of cucurbitaceae plants[6,7] have been shown to possess antiulcer and antioxidant activity, viz. *Cucurbita moschata* (Fruit), *Momordica charantia* (Immature fruits), *Cucumis melo* (Mature fruit), etc. Hence, the objective of the present study was to evaluate gastroprotective effect of methanol and petroleum ether extracts of *B. hispida* fruit using different experimental models.

## Materials and Methods

### Collection of plant

The fresh fruit of *B. hispida* was collected in the month of September 2004 from the local vegetable market of Ahmedabad, Gujarat. The authentification of the plant was done in the department of Pharmacognosy, L. M. College of Pharmacy, Ahmedabad, Gujarat, India.

### Extraction procedure

The fruit was dried, seeds were separated and powdered, and passed to 60 mesh. The powder of fruit (500 g) was first defatted with petroleum ether (500 ml × 6; yield was 1% w/w). The defatted powder was then dried and extracted with methanol (500 ml × 6; yield was 20% w/w) and thereafter with ethyl acetate (500 ml × 4; yield was 3% w/w) successively. The remaining powder was dried and extracted with distilled water to give aqueous extract (500 ml × 6; yield was 5% w/w). The dried fractions were stored in a refrigerator at 4°C throughout the study.

Preliminary thin layer chromatography (TLC) study was carried out for checking the presence of phytoconstituents as beta-sitosterol, lupeol, iso-vitexin, iso-multiflorenol, cucurbitacin-B, different amino acids, etc. which have been reported to be present in the fruit of *B. hispida*. Further, quantitative estimation of phytoconstituents using high pressure thin layer chromatography (HPTLC) fingerprinting was also carried out for both the extracts using RP-18 silica, briefed as follows:

1. Betasitosterol in petroleum ether extract (PEBH)
  Petroleum ether: Acetonitrile: Methanol (1:2:2)
2. Lupeol in PEBH
  Benzene: Ethyl acetate (9.5: 0.5)
3. Iso-vitexin in methanol extract (MEBH)
  Ethyl acetate: n-butanol: Water (2:1:3)

### Animals

Wistar albino rats of either sex, weighing between 200–250 g were fed with standard chow diet. All animals were kept under a controlled light/dark cycle and temperature (22 ± 2°C) with free access to food and water. This experiment complied with the guidelines for animal experimentation of our laboratory and approved by Institutional Animal Ethics Committee (IAEC).

### Chemicals

Absolute alcohol (Alembic Chemicals Works, India), omeprazole (Zydus Research Centre, India), Evan's blue (Qualigens, India), thiobarbituric acid (Sigma Aldrich, USA), adrenaline bitarterate (Camphossa, India), and hydrogen peroxide (S.D. Fine Chemicals Pvt. Ltd, India) were used in the experiment.

### Experimental design

The animals were divided into following groups of six animals each. Preliminary study using different doses of petroleum ether, methanol, ethyl acetate, and aqueous extracts against ethanol-induced gastric mucosal damage revealed significant protection at the dose of 300 mg/kg (p.o.). Further study was carried out using petroleum ether (PEBH) and methanol extracts (MEBH) as they showed marked protection against gastric lesions as compared to other extracts.

- Group 1 (control): Animals received only aqueous suspension of 1% w/v sodium carboxy methyl cellulose (Sod. CMC) as vehicle.
- Group 2 (test 1): Animals received PEBH (300 mg/kg, p.o.).
- Group 3 (test 2): Animals received MEBH (300 mg/kg, p.o.).
- Group 4 (standard): Animals received omeprazole (20 mg/kg, p.o.).

### Ethanol-induced gastric mucosal damage[8]

In addition, ethyl acetate (300 mg/kg, p.o.) and aqueous extract (300 mg/kg, p.o.) were also tested in this model. Animals were fasted for 16 h before an experiment but were allowed free access to water. One milliliter of absolute ethanol was administered orally to rats. In treatment group, drugs were administered orally 1 h before the administration of ethanol. After 2 h of ethanol treatment, animals were sacrificed; stomachs were removed, opened along the greater curvature, and examined for lesions. Lesion severity was determined by measuring ulcer index.[9] Additionally, vascular permeability was determined in case of MEBH and PEBH treated groups.[10]

### Pylorus ligated ulcer model[11]

Rats (16-h fasted) were anesthetized with light ether anesthesia. A portion of the abdomen was opened by a small incision below the xiphoid process. Pyloric portion of the stomach was slightly lifted out avoiding traction to the pylorus or damage to its blood supply. The stomach was replaced carefully and interrupted sutures closed the abdominal wall. The drugs were administered orally and animals were sacrificed at the end of 6 h after operation. Stomach was dissected out; contents were drained into tubes and subjected to analysis for different physical parameters as well as acid secretory and mucoprotective parameters.[12]

### Cold and restraint stress-induced gastric ulcer model[13]

In this method, Wistar rats were deprived of food for 12 h. They were then immobilized in a restrainer (stress cage) and forced to remain in refrigerator (4–6°C) for 3 h. The animals were sacrificed using high dose of ether and ulcer-index was calculated. The test drug was administered orally 30 min before immobilizing the animals. In this model, free radical levels were also tested to determine the extent of lipid peroxidation, viz. melondialdehyde (MDA),[14] superoxide dismutase (SOD)[15] and catalase (CAT).[16]

### Statistical analysis

The results were expressed in terms of mean ± SEM. Significance was determined by one-way analysis of variance followed by the Tukey's multiple range test. Statistical analysis was performed using statistical analysis software Sigma Stat 2.0. In each case *P* values less than 0.05 were considered as indicative of significance.

## Results

Thin layer chromatographic study suggested the presence of beta-sitosterol and lupeol in PEBH and presence of iso-vitexin in case of MEBH.

High pressure thin layer chromatography study revealed the presence of 6.52% w/w of beta-sitosterol and 2.48% w/w of lupeol in PEBH which are equivalents to 0.013% w/w and 0.0049% w/w in dry powder, respectively. The level of iso-vitexin in MEBH was 0.85% w/w which is equivalent to 0.17% w/w in dry powder.

### Ethanol-induced gastric mucosal damage

**Figure 1:**
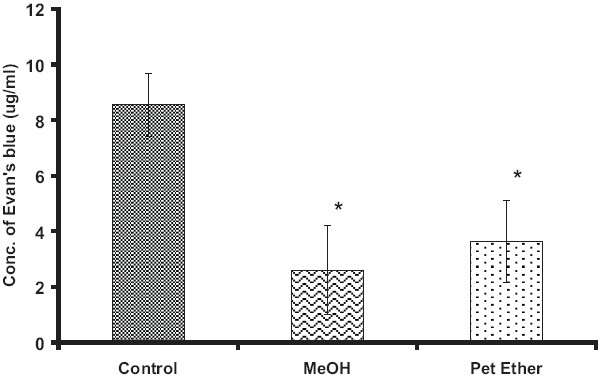
Effect of *Benincasa hispida* on ethanol-induced vascular permeability. All values represent Mean ± SEM, n = 6 in each group. *P < 0.05, when compared with the control group (ANOVA, followed by Tukey's multiple range test)

Different extracts of *B. hispida*, viz. PEBH, MEBH, and ethyl acetate extract (300 mg/kg, p.o.) showed significant reduction (*P* < 0.05) in ulcer index when compared with the control group and results were comparable with that of omeprazole-treated rats [Table 1]. However, reduction in ulcer index with aqueous extract pretreatment was not found to be statistically significant. Further, PEBH and MEBH pretreatment resulted in significant reduction in vascular permeability [Figure 1].

**Table 1:** Effect of different extracts of ***Benincasa hispida*** on ethanol-induced, pylorus ligated, and cold related stress model in rats

*Group (mg/kg, p.o.)*	*Ethanol-model*	*Ulcer index (% protection) PL-model*	*CRS-model*
Control (1% w/v Sod. CMC)	1.51 ± 0.152	1.83 ± 0.192	0.674 ± 0.040
(5 ml/kg, p.o.)	(0.0)	(0.0)	(0.0)
Petroleum ether extract	0.76 ± 0.056*	0.27 ± 0.068*	0.255 ± 0.056*
(300)	(49.03)	(85.26)	(62.13)
Methanol extract	0.49 ± 0.096*	0.44 ± 0.086*	0.327 ± 0.041*
(300)	(67.36)	(75.96)	(51.52)
Ethyl acetate extract	0.95 ± 0.110*	----	----
(300)	(36.91)		
Aqueous extract	1.17 ± 0.230	----	----
(300)	(22.30)		
Omeprazole	0.58 ± 0.075*	0.39 ± 0.126*	0.351 ± 0.030*
(20)	(61.26)	(78.69)	(47.83)

All values represent mean ± SEM, n = 6 in each group.

* *P* < 0.05, when compared with the control group (ANOVA, followed by Tukey's multiple range test)

### Pylorus ligation model

PEBH and MEBH treated animals showed significant reduction (*P* < 0.05) in ulcer index [Table 1]. MEBH and omeprazole treatment showed significant reduction (*P* < 0.05) in volume of acid secretion, acid output, pepsin activity, and protein content along with significant increase in pH, carbohydrate content, and TC:PR ratio (mucin activity). PEBH showed significant reduction (*P* < 0.05) in pepsin activity and protein content along with increase in carbohydrate content and TC:PR ratio (mucin activity) [Table 2].

**Table 2:** Effect of PEBH and MEBH extracts† of ***Benincasa hispida*** on acid secretary parameters in pylorus ligated gastric ulcer model

*Group (mg/kg, p.o.)*	*Vol. of gas content (ml/100 g b.w.)(pH)*	*Total acidity(m/Eql)*	*Acid output(mEq/100 g b.w.)*	*Pepsin activity (μg/ml)*	*Total carbohydrates (TC) (mg/ml)*	*Protein content (PR) (mg/ml)*	*Mucin activity (TC/PR) ratio*
Control (1% w/v Sod. CMC)	3.44 ± 0.362	3.83 ± 0.461	13.16 ± 0.407	15.42 ± 1.842	0.658 ± 0.159	0.608 ± 0.087	1.082 ± 0.241
(5 ml/kg, p.o.)	(4.67 ± 0.272)						
Petroleum ether extract	3.04 ± 0.786	4.58 ± 0.057	13.92 ± 0.109	2.33 ± 1.094*	3.240 ± 0.348*	0.627 ± 0.038	5.169± 0.461*
	(4.67 ± 0.544)						
Methanol extract	1.32 ± 0.424*	4.13 ± 0.253	5.45 ± 0.262*	3.75 ± 1.470*	5.225 ± 0.726*	0.250± 0.064*	20.9 ± 1.943*
	(6.38± 0.210*)						
Omeprazole (20)	1.29 ± 0.355*	3.77 ± 0.567	4.86 ± 0.487*	5.45 ± 1.226*	0.482 ± 0.046	0.321± 0.058*	1.502 ± 0.035
	(6.25± 0.241*)						

All values represent mean ± SEM, n = 6 in each group.

† 300 mg/kg;

* *P* < 0.05, when compared with the control group (ANOVA, followed by Tukey's multiple range test)

### Cold restraint stress-induced gastric ulcer model

PEBH and MEBH treatment resulted in significant reduction in ulcer index and MDA contents along with increase in the CAT levels (*P* < 0.05). However, no significant effect was observed on SOD levels [Tables 1 and 3].

**Table 3:** Effect of PEBH and MEBH extracts of ***Benincasa hispida*** on MDA content and antioxidant enzymes against cold restraint stress-induced gastric ulcer model

*Group*	*Dose (mg/kg, p.o.)*	*MDA content (μmole/mg protein)*	*SOD(Units/min/mg protein)*	*CAT(Units/min/mg protein)*
Control (1% w/v Sod. CMC)	5 ml	0.740 ± 0.056	0.116 ± 0.007	2.88 ± 0.217
Petroleum ether extract	300	0.307 ± 0.002*	0.126 ± 0.002	4.39 ± 0.341*
Methanol extract	300	0.308 ± 0.025*	0.140 ± 0.010	5.18 ± 0.273*
Omeprazole	20	0.471 ± 0.116*	0.211 ± 0.005*	4.93 ± 0.200*

All values represent Mean ± SEM, n = 6 in each group.

* *P* < 0.05, when compared with the control group (ANOVA, followed by Tukey's multiple range test)

## Discussions

The observations of the present study suggest significant antiulcer activity of PEBH and MEBH of *B. hispida* against ethanol-, CRS-, and PL- induced gastric ulcers in rats.

Ethanol and several NSAIDS, such as aspirin, irritate the gastrointestinal mucosa in both human and animals and may, therefore, cause injury and bleeding. Ulcers caused by chemical inducers like ethanol are due to a number of contributing factors, which include effects on mucosal blood flow, platelet thrombi, damage to capillary endothelium, and release of arachidonate metabolites, leukotriene C4/D4 (LTC4/D4), and platelet activating factor (PAF).[17] Involvement of free radicals is also reported for gastric ulceration caused by ethanol.[18] Thus, the protection afforded by MEBH and PEBH in ethanol model can be correlated to decrease in vascular permeability and thereby preventing damage to the capillary endothelium and release of arachidonate metabolites. However, MEBH showed better protection (67%) than PEBH (49%) which could be due to its marked antioxidant activity as shown in stress model.

Pylorus ligated induced ulcers are thought to be caused due to increased presence of acid and pepsin in the stomach. The essential criteria, which determine the status of mucosal defense barrier against the offensive assault of acid-pepsin is the quality and quantity of gastric mucus secretion.[19] Increased mucus secretion by the gasric mucosal cells can prevent gasric ulceration by several mechanisms including lessening stomach wall friction during peristalsis and acting as an effective barrier to the back diffusion of hydrogen ions. The TC/PR ratio has been accepted as a reliable index of mucosal resistance.[19] MEBH pretreatment have shown significant reduction in protein levels with corresponding increase in carbohydrate level leading to marked rise in mucin activity, although PEBH extract also significantly increased carbohydrate level with no change in protein levels. Hence, the protection afforded by MEBH and PEBH against gastric ulcers induced by PL appears to be produced by the suppression of pepsin levels and strengthening of mucosal barrier. Further, it is clear that the antiulcer activity against this model was again found to be more with MEBH extract.

Stress-induced ulcers are caused by a number of factors both physical and psychological.[20] Increase in gastric motility, vagal overactivity, mast cell degranulation, decreased mucosal blood flow, and decreased PG synthesis are reported to be involved in the genesis of stress-induced ulcers.[21–23] Free radicals are also involved in the gastric ulcer caused by stress. In stress-induced gastric ulceration, MDA levels significantly increases with concomitant decrease in SOD and CAT concentration as reported by Das and coworkers.[24] Increase in MDA levels results into increase in reactive oxygen species (ROS), the major radicals being superoxide anion, H_2_O_2_, and hydroxyl radical. These induce cell degranulation by increasing peroxidation of cell membrane lipids, causing loss of structural and functional integrity of cell membranes. Accumulation of the H_2_O_2_ occurs in the mitochondria and cytosol, if not scavenged by CAT leads to increase in generation of OH^−^ radical. Thus, decreased CAT levels lead to increased lipid peroxidation.[25] As shown in the results, PEBH and MEBH treatment significantly reverted the stress-induced changes in MDA and CAT, with no remarkable change in SOD activities. This reduction in MDA levels (*P* < 0.05) along with significant increase in CAT levels strongly suggest decreased lipid peroxidation and antioxidant activity of PEBH and MEBH.

The present study reports the antiulcerogenic and antioxidant effect of petroleum ether and methanol extracts of *B. hispida*. The mechanism of its gastroprotective activity may be attributed to reduction in vascular permeability, free radical generation, and lipid peroxidation along with strengthening of mucosal barrier. Besides, presence of phytoconstituents in this plant like flavone and sterols might be responsible for these actions. Our future study will be directed to identify the phytoconstituents responsible for these pharmacological actions of *B. hispida* fruit.
